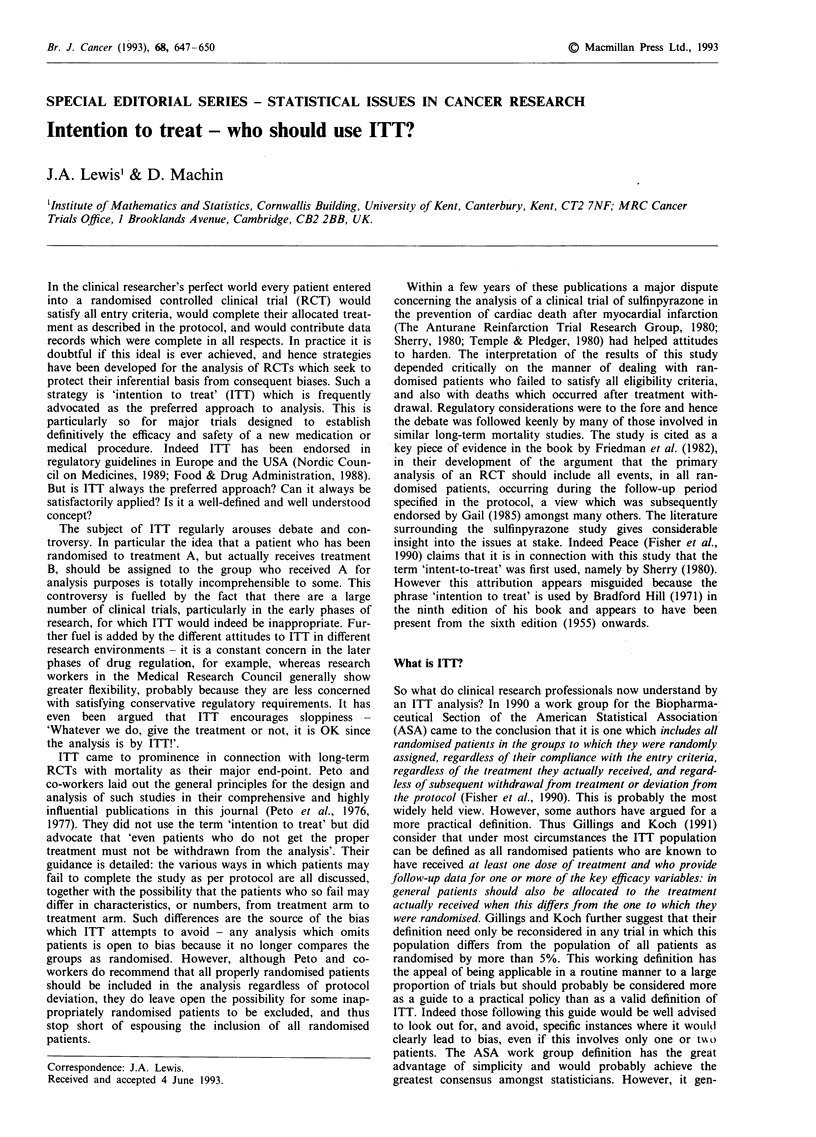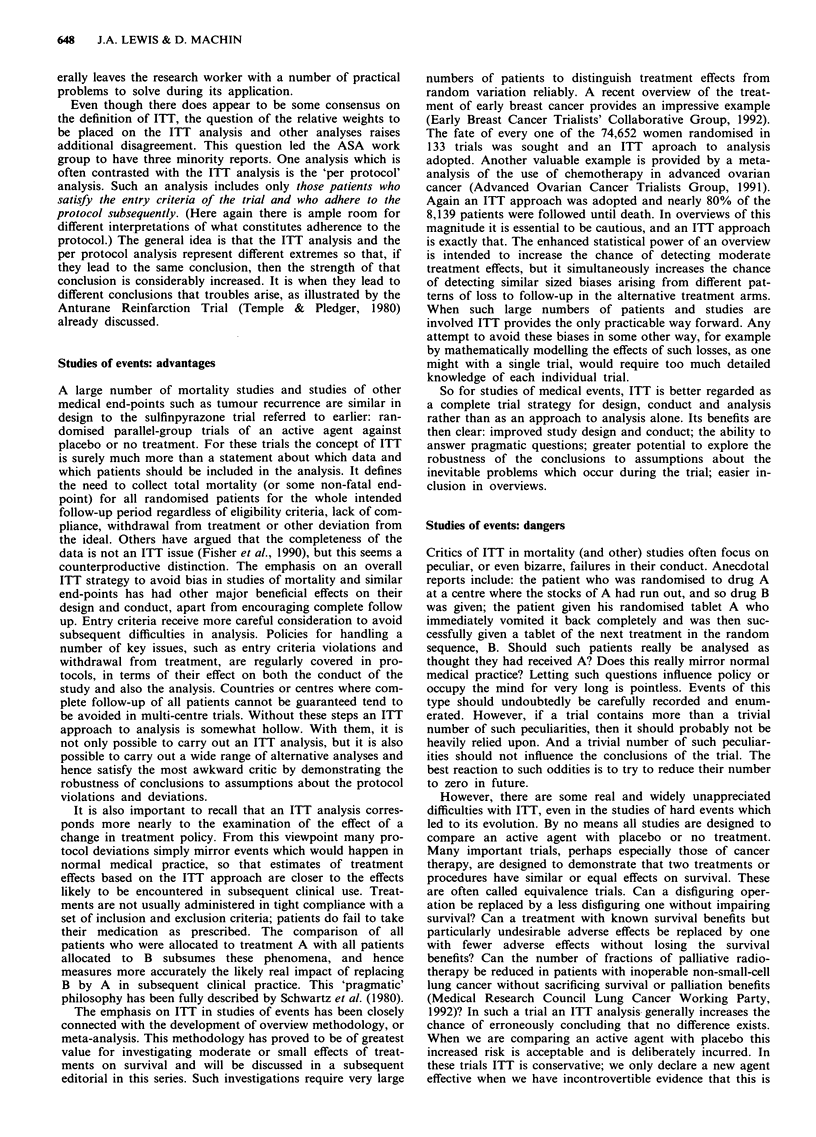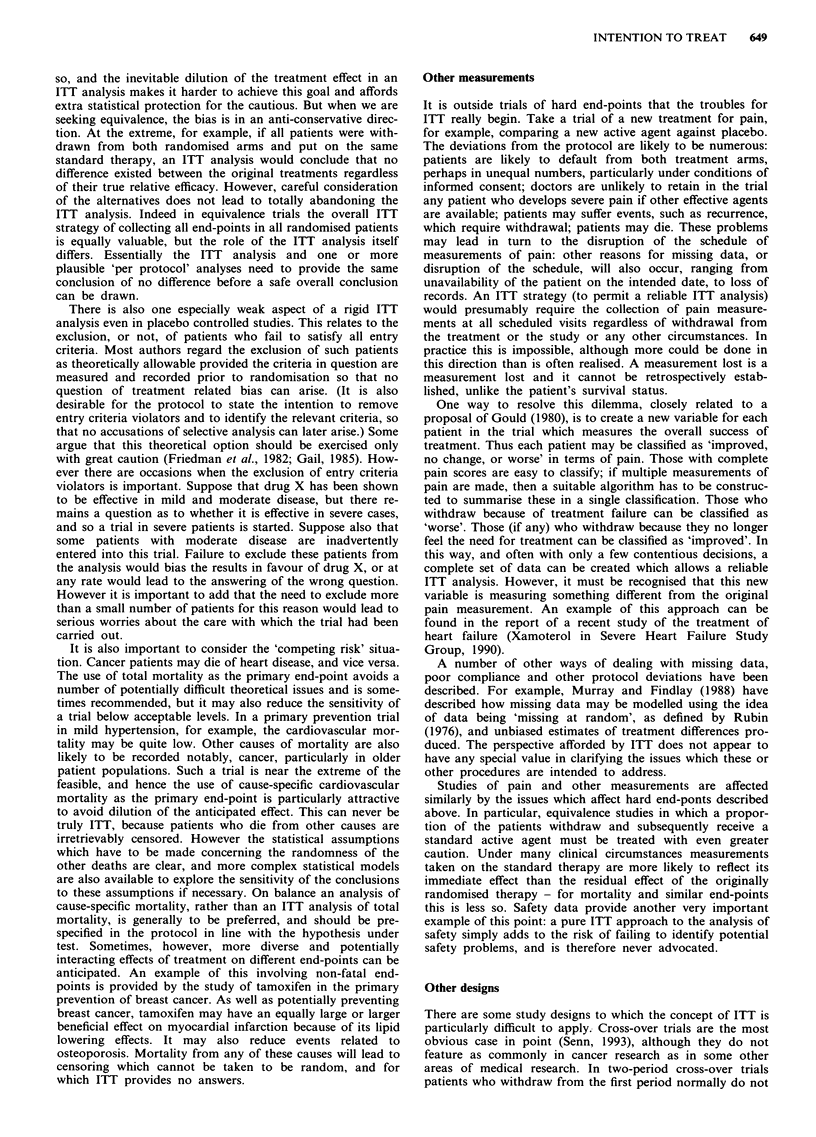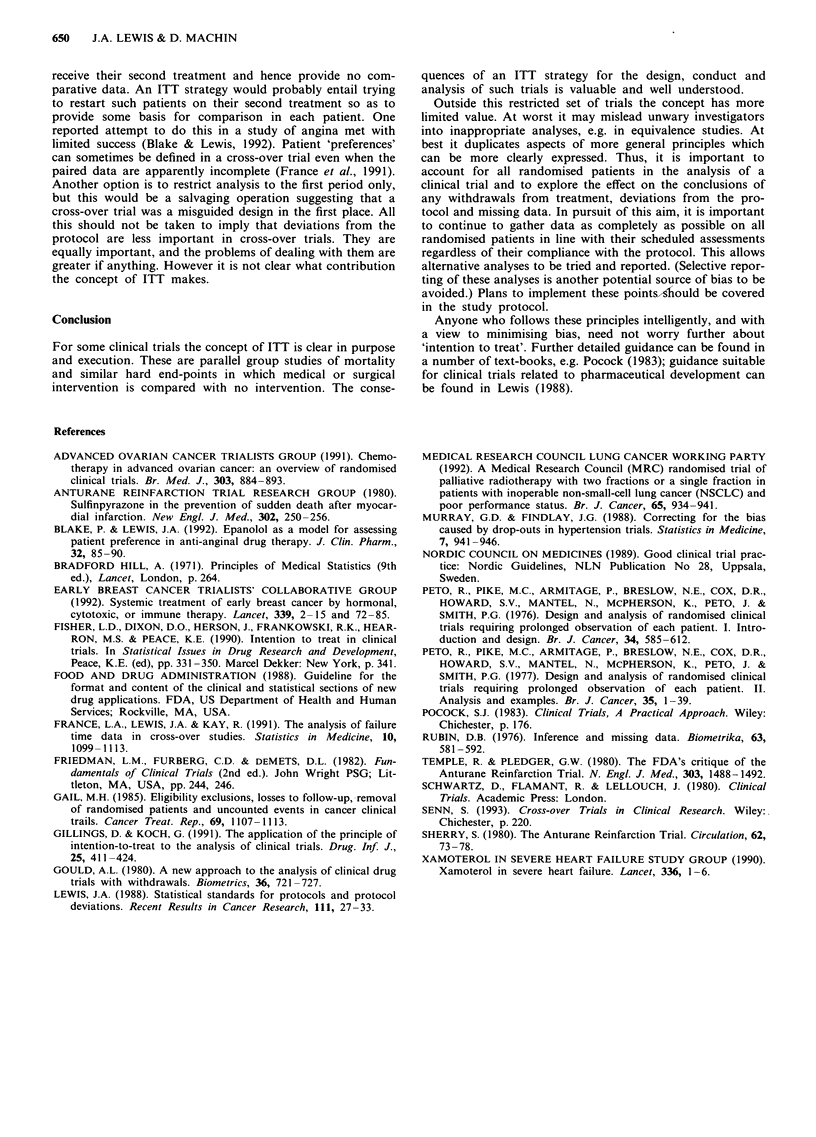# Intention to treat--who should use ITT?

**DOI:** 10.1038/bjc.1993.402

**Published:** 1993-10

**Authors:** J. A. Lewis, D. Machin


Br. J. Cancer (1993), 68, 647–650 © Macmillan Press Ltd., 1993

SPECIAL EDITORIAL SERIES – STATISTICAL ISSUES IN CANCER RESEARCH

### Intention to treat – who should use ITT?

J.A. Lewis[1] & D. Machin

[1]*Institute of Mathematics and Statistics, Cornwallis Building, University of Kent, Canterbury, Kent, CT2 7NF; MRC Cancer Trials Office, 1 Brooklands Avenue, Cambridge, CB2 2BB, UK.*

In the clinical researcher's perfect world every patient entered into a randomised controlled clinical trial (RCT) would satisfy all entry criteria, would complete their allocated treatment as described in the protocol, and would contribute data records which were complete in all respects. In practice it is doubtful if this ideal is ever achieved, and hence strategies have been developed for the analysis of RCTs which seek to protect their inferential basis from consequent biases. Such a strategy is 'intention to treat' (ITT) which is frequently advocated as the preferred approach to analysis. This is particularly so for major trials designed to establish definitively the efficacy and safety of a new medication or medical procedure. Indeed ITT has been endorsed in regulatory guidelines in Europe and the USA (Nordic Council on Medicines, 1989; Food & Drug Administration, 1988). But is ITT always the preferred approach? Can it always be satisfactorily applied? Is it a well-defined and well understood concept?

The subject of ITT regularly arouses debate and controversy. In particular the idea that a patient who has been randomised to treatment A, but actually receives treatment B, should be assigned to the group who received A for analysis purposes is totally incomprehensible to some. This controversy is fuelled by the fact that there are a large number of clinical trials, particularly in the early phases of research, for which ITT would indeed be inappropriate. Further fuel is added by the different attitudes to ITT in different research environments – it is a constant concern in the later phases of drug regulation, for example, whereas research workers in the Medical Research Council generally show greater flexibility, probably because they are less concerned with satisfying conservative regulatory requirements. It has even been argued that ITT encourages sloppiness – 'Whatever we do, give the treatment or not, it is OK since the analysis is by ITT!'.

ITT came to prominence in connection with long-term RCTs with mortality as their major end-point. Peto and co-workers laid out the general principles for the design and analysis of such studies in their comprehensive and highly influential publications in this journal (Peto *et al.*, 1976, 1977). They did not use the term 'intention to treat' but did advocate that 'even patients who do not get the proper treatment must not be withdrawn from the analysis'. Their guidance is detailed: the various ways in which patients may fail to complete the study as per protocol are all discussed, together with the possibility that the patients who so fail may differ in characteristics, or numbers, from treatment arm to treatment arm. Such differences are the source of the bias which ITT attempts to avoid – any analysis which omits patients is open to bias because it no longer compares the groups as randomised. However, although Peto and co-workers do recommend that all properly randomised patients should be included in the analysis regardless of protocol deviation, they do leave open the possibility for some inappropriately randomised patients to be excluded, and thus stop short of espousing the inclusion of all randomised patients.

Correspondence: J.A. Lewis.
Received and accepted 4 June 1993.

Within a few years of these publications a major dispute concerning the analysis of a clinical trial of sulfinpyrazone in the prevention of cardiac death after myocardial infarction (The Anturane Reinfarction Trial Research Group, 1980; Sherry, 1980; Temple & Pledger, 1980) had helped attitudes to harden. The interpretation of the results of this study depended critically on the manner of dealing with randomised patients who failed to satisfy all eligibility criteria, and also with deaths which occurred after treatment withdrawal. Regulatory considerations were to the fore and hence the debate was followed keenly by many of those involved in similar long-term mortality studies. The study is cited as a key piece of evidence in the book by Friedman *et al.* (1982), in their development of the argument that the primary analysis of an RCT should include all events, in all randomised patients, occurring during the follow-up period specified in the protocol, a view which was subsequently endorsed by Gail (1985) amongst many others. The literature surrounding the sulfinpyrazone study gives considerable insight into the issues at stake. Indeed Peace (Fisher *et al.*, 1990) claims that it is in connection with this study that the term 'intent-to-treat' was first used, namely by Sherry (1980). However this attribution appears misguided because the phrase 'intention to treat' is used by Bradford Hill (1971) in the ninth edition of his book and appears to have been present from the sixth edition (1955) onwards.

### What is ITT?

So what do clinical research professionals now understand by an ITT analysis? In 1990 a work group for the Biopharmaceutical Section of the American Statistical Association (ASA) came to the conclusion that it is one which *includes all randomised patients in the groups to which they were randomly assigned, regardless of their compliance with the entry criteria, regardless of the treatment they actually received, and regardless of subsequent withdrawal from treatment or deviation from the protocol* (Fisher *et al.*, 1990). This is probably the most widely held view. However, some authors have argued for a more practical definition. Thus Gillings and Koch (1991) consider that under most circumstances the ITT population can be defined as all randomised patients who are known to have received *at least one dose of treatment and who provide follow-up data for one or more of the key efficacy variables: in general patients should also be allocated to the treatment actually received when this differs from the one to which they were randomised.* Gillings and Koch further suggest that their definition need only be reconsidered in any trial in which this population differs from the population of all patients as randomised by more than 5%. This working definition has the appeal of being applicable in a routine manner to a large proportion of trials but should probably be considered more as a guide to a practical policy than as a valid definition of ITT. Indeed those following this guide would be well advised to look out for, and avoid, specific instances where it would clearly lead to bias, even if this involves only one or two patients. The ASA work group definition has the great advantage of simplicity and would probably achieve the greatest consensus amongst statisticians. However, it generally leaves the research worker with a number of practical problems to solve during its application.

Even though there does appear to be some consensus on the definition of ITT, the question of the relative weights to be placed on the ITT analysis and other analyses raises additional disagreement. This question led the ASA work group to have three minority reports. One analysis which is often contrasted with the ITT analysis is the 'per protocol' analysis. Such an analysis includes only *those patients who satisfy the entry criteria of the trial and who adhere to the protocol subsequently*. (Here again there is ample room for different interpretations of what constitutes adherence to the protocol.) The general idea is that the ITT analysis and the per protocol analysis represent different extremes so that, if they lead to the same conclusion, then the strength of that conclusion is considerably increased. It is when they lead to different conclusions that troubles arise, as illustrated by the Anturane Reinfarction Trial (Temple & Pledger, 1980) already discussed.

### Studies of events: advantages

A large number of mortality studies and studies of other medical end-points such as tumour recurrence are similar in design to the sulfinpyrazone trial referred to earlier: randomised parallel-group trials of an active agent against placebo or no treatment. For these trials the concept of ITT is surely much more than a statement about which data and which patients should be included in the analysis. It defines the need to collect total mortality (or some non-fatal end-point) for all randomised patients for the whole intended follow-up period regardless of eligibility criteria, lack of compliance, withdrawal from treatment or other deviation from the ideal. Others have argued that the completeness of the data is not an ITT issue (Fisher *et al*., 1990), but this seems a counterproductive distinction. The emphasis on an overall ITT strategy to avoid bias in studies of mortality and similar end-points has had other major beneficial effects on their design and conduct, apart from encouraging complete follow up. Entry criteria receive more careful consideration to avoid subsequent difficulties in analysis. Policies for handling a number of key issues, such as entry criteria violations and withdrawal from treatment, are regularly covered in protocols, in terms of their effect on both the conduct of the study and also the analysis. Countries or centres where complete follow-up of all patients cannot be guaranteed tend to be avoided in multi-centre trials. Without these steps an ITT approach to analysis is somewhat hollow. With them, it is not only possible to carry out an ITT analysis, but it is also possible to carry out a wide range of alternative analyses and hence satisfy the most awkward critic by demonstrating the robustness of conclusions to assumptions about the protocol violations and deviations.

It is also important to recall that an ITT analysis corresponds more nearly to the examination of the effect of a change in treatment policy. From this viewpoint many protocol deviations simply mirror events which would happen in normal medical practice, so that estimates of treatment effects based on the ITT approach are closer to the effects likely to be encountered in subsequent clinical use. Treatments are not usually administered in tight compliance with a set of inclusion and exclusion criteria; patients do fail to take their medication as prescribed. The comparison of all patients who were allocated to treatment A with all patients allocated to B subsumes these phenomena, and hence measures more accurately the likely real impact of replacing B by A in subsequent clinical practice. This 'pragmatic' philosophy has been fully described by Schwartz *et al*. (1980).

The emphasis on ITT in studies of events has been closely connected with the development of overview methodology, or meta-analysis. This methodology has proved to be of greatest value for investigating moderate or small effects of treatments on survival and will be discussed in a subsequent editorial in this series. Such investigations require very large numbers of patients to distinguish treatment effects from random variation reliably. A recent overview of the treatment of early breast cancer provides an impressive example (Early Breast Cancer Trialists' Collaborative Group, 1992). The fate of every one of the 74,652 women randomised in 133 trials was sought and an ITT aproach to analysis adopted. Another valuable example is provided by a meta-analysis of the use of chemotherapy in advanced ovarian cancer (Advanced Ovarian Cancer Trialists Group, 1991). Again an ITT approach was adopted and nearly 80% of the 8,139 patients were followed until death. In overviews of this magnitude it is essential to be cautious, and an ITT approach is exactly that. The enhanced statistical power of an overview is intended to increase the chance of detecting moderate treatment effects, but it simultaneously increases the chance of detecting similar sized biases arising from different patterns of loss to follow-up in the alternative treatment arms. When such large numbers of patients and studies are involved ITT provides the only practicable way forward. Any attempt to avoid these biases in some other way, for example by mathematically modelling the effects of such losses, as one might with a single trial, would require too much detailed knowledge of each individual trial.

So for studies of medical events, ITT is better regarded as a complete trial strategy for design, conduct and analysis rather than as an approach to analysis alone. Its benefits are then clear: improved study design and conduct; the ability to answer pragmatic questions; greater potential to explore the robustness of the conclusions to assumptions about the inevitable problems which occur during the trial; easier inclusion in overviews.

### Studies of events: dangers

Critics of ITT in mortality (and other) studies often focus on peculiar, or even bizarre, failures in their conduct. Anecdotal reports include: the patient who was randomised to drug A at a centre where the stocks of A had run out, and so drug B was given; the patient given his randomised tablet A who immediately vomited it back completely and was then successfully given a tablet of the next treatment in the random sequence, B. Should such patients really be analysed as thought they had received A? Does this really mirror normal medical practice? Letting such questions influence policy or occupy the mind for very long is pointless. Events of this type should undoubtedly be carefully recorded and enumerated. However, if a trial contains more than a trivial number of such peculiarities, then it should probably not be heavily relied upon. And a trivial number of such peculiarities should not influence the conclusions of the trial. The best reaction to such oddities is to try to reduce their number to zero in future.

However, there are some real and widely unappreciated difficulties with ITT, even in the studies of hard events which led to its evolution. By no means all studies are designed to compare an active agent with placebo or no treatment. Many important trials, perhaps especially those of cancer therapy, are designed to demonstrate that two treatments or procedures have similar or equal effects on survival. These are often called equivalence trials. Can a disfiguring operation be replaced by a less disfiguring one without impairing survival? Can a treatment with known survival benefits but particularly undesirable adverse effects be replaced by one with fewer adverse effects without losing the survival benefits? Can the number of fractions of palliative radiotherapy be reduced in patients with inoperable non-small-cell lung cancer without sacrificing survival or palliation benefits (Medical Research Council Lung Cancer Working Party, 1992)? In such a trial an ITT analysis generally increases the chance of erroneously concluding that no difference exists. When we are comparing an active agent with placebo this increased risk is acceptable and is deliberately incurred. In these trials ITT is conservative; we only declare a new agent effective when we have incontrovertible evidence that this is so, and the inevitable dilution of the treatment effect in an ITT analysis makes it harder to achieve this goal and affords extra statistical protection for the cautious. But when we are seeking equivalence, the bias is in an anti-conservative direction. At the extreme, for example, if all patients were withdrawn from both randomised arms and put on the same standard therapy, an ITT analysis would conclude that no difference existed between the original treatments regardless of their true relative efficacy. However, careful consideration of the alternatives does not lead to totally abandoning the ITT analysis. Indeed in equivalence trials the overall ITT strategy of collecting all end-points in all randomised patients is equally valuable, but the role of the ITT analysis itself differs. Essentially the ITT analysis and one or more plausible 'per protocol' analyses need to provide the same conclusion of no difference before a safe overall conclusion can be drawn.

There is also one especially weak aspect of a rigid ITT analysis even in placebo controlled studies. This relates to the exclusion, or not, of patients who fail to satisfy all entry criteria. Most authors regard the exclusion of such patients as theoretically allowable provided the criteria in question are measured and recorded prior to randomisation so that no question of treatment related bias can arise. (It is also desirable for the protocol to state the intention to remove entry criteria violators and to identify the relevant criteria, so that no accusations of selective analysis can later arise.) Some argue that this theoretical option should be exercised only with great caution (Friedman *et al.*, 1982; Gail, 1985). However there are occasions when the exclusion of entry criteria violators is important. Suppose that drug X has been shown to be effective in mild and moderate disease, but there remains a question as to whether it is effective in severe cases, and so a trial in severe patients is started. Suppose also that some patients with moderate disease are inadvertently entered into this trial. Failure to exclude these patients from the analysis would bias the results in favour of drug X, or at any rate would lead to the answering of the wrong question. However it is important to add that the need to exclude more than a small number of patients for this reason would lead to serious worries about the care with which the trial had been carried out.

It is also important to consider the 'competing risk' situation. Cancer patients may die of heart disease, and vice versa. The use of total mortality as the primary end-point avoids a number of potentially difficult theoretical issues and is sometimes recommended, but it may also reduce the sensitivity of a trial below acceptable levels. In a primary prevention trial in mild hypertension, for example, the cardiovascular mortality may be quite low. Other causes of mortality are also likely to be recorded notably, cancer, particularly in older patient populations. Such a trial is near the extreme of the feasible, and hence the use of cause-specific cardiovascular mortality as the primary end-point is particularly attractive to avoid dilution of the anticipated effect. This can never be truly ITT, because patients who die from other causes are irretrievably censored. However the statistical assumptions which have to be made concerning the randomness of the other deaths are clear, and more complex statistical models are also available to explore the sensitivity of the conclusions to these assumptions if necessary. On balance an analysis of cause-specific mortality, rather than an ITT analysis of total mortality, is generally to be preferred, and should be prespecified in the protocol in line with the hypothesis under test. Sometimes, however, more diverse and potentially interacting effects of treatment on different end-points can be anticipated. An example of this involving non-fatal end-points is provided by the study of tamoxifen in the primary prevention of breast cancer. As well as potentially preventing breast cancer, tamoxifen may have an equally large or larger beneficial effect on myocardial infarction because of its lipid lowering effects. It may also reduce events related to osteoporosis. Mortality from any of these causes will lead to censoring which cannot be taken to be random, and for which ITT provides no answers.

### Other measurements

It is outside trials of hard end-points that the troubles for ITT really begin. Take a trial of a new treatment for pain, for example, comparing a new active agent against placebo. The deviations from the protocol are likely to be numerous: patients are likely to default from both treatment arms, perhaps in unequal numbers, particularly under conditions of informed consent; doctors are unlikely to retain in the trial any patient who develops severe pain if other effective agents are available; patients may suffer events, such as recurrence, which require withdrawal; patients may die. These problems may lead in turn to the disruption of the schedule of measurements of pain: other reasons for missing data, or disruption of the schedule, will also occur, ranging from unavailability of the patient on the intended date, to loss of records. An ITT strategy (to permit a reliable ITT analysis) would presumably require the collection of pain measurements at all scheduled visits regardless of withdrawal from the treatment or the study or any other circumstances. In practice this is impossible, although more could be done in this direction than is often realised. A measurement lost is a measurement lost and it cannot be retrospectively established, unlike the patient's survival status.

One way to resolve this dilemma, closely related to a proposal of Gould (1980), is to create a new variable for each patient in the trial which measures the overall success of treatment. Thus each patient may be classified as 'improved, no change, or worse' in terms of pain. Those with complete pain scores are easy to classify; if multiple measurements of pain are made, then a suitable algorithm has to be constructed to summarise these in a single classification. Those who withdraw because of treatment failure can be classified as 'worse'. Those (if any) who withdraw because they no longer feel the need for treatment can be classified as 'improved'. In this way, and often with only a few contentious decisions, a complete set of data can be created which allows a reliable ITT analysis. However, it must be recognised that this new variable is measuring something different from the original pain measurement. An example of this approach can be found in the report of a recent study of the treatment of heart failure (Xamoterol in Severe Heart Failure Study Group, 1990).

A number of other ways of dealing with missing data, poor compliance and other protocol deviations have been described. For example, Murray and Findlay (1988) have described how missing data may be modelled using the idea of data being 'missing at random', as defined by Rubin (1976), and unbiased estimates of treatment differences produced. The perspective afforded by ITT does not appear to have any special value in clarifying the issues which these or other procedures are intended to address.

Studies of pain and other measurements are affected similarly by the issues which affect hard end-ponts described above. In particular, equivalence studies in which a proportion of the patients withdraw and subsequently receive a standard active agent must be treated with even greater caution. Under many clinical circumstances measurements taken on the standard therapy are more likely to reflect its immediate effect than the residual effect of the originally randomised therapy – for mortality and similar end-points this is less so. Safety data provide another very important example of this point: a pure ITT approach to the analysis of safety simply adds to the risk of failing to identify potential safety problems, and is therefore never advocated.

### Other designs

There are some study designs to which the concept of ITT is particularly difficult to apply. Cross-over trials are the most obvious case in point (Senn, 1993), although they do not feature as commonly in cancer research as in some other areas of medical research. In two-period cross-over trials patients who withdraw from the first period normally do not receive their second treatment and hence provide no comparative data. An ITT strategy would probably entail trying to restart such patients on their second treatment so as to provide some basis for comparison in each patient. One reported attempt to do this in a study of angina met with limited success (Blake & Lewis, 1992). Patient 'preferences' can sometimes be defined in a cross-over trial even when the paired data are apparently incomplete (France *et al.*, 1991). Another option is to restrict analysis to the first period only, but this would be a salvaging operation suggesting that a cross-over trial was a misguided design in the first place. All this should not be taken to imply that deviations from the protocol are less important in cross-over trials. They are equally important, and the problems of dealing with them are greater if anything. However it is not clear what contribution the concept of ITT makes.

### Conclusion

For some clinical trials the concept of ITT is clear in purpose and execution. These are parallel group studies of mortality and similar hard end-points in which medical or surgical intervention is compared with no intervention. The consequences of an ITT strategy for the design, conduct and analysis of such trials is valuable and well understood.

Outside this restricted set of trials the concept has more limited value. At worst it may mislead unwary investigators into inappropriate analyses, e.g. in equivalence studies. At best it duplicates aspects of more general principles which can be more clearly expressed. Thus, it is important to account for all randomised patients in the analysis of a clinical trial and to explore the effect on the conclusions of any withdrawals from treatment, deviations from the protocol and missing data. In pursuit of this aim, it is important to continue to gather data as completely as possible on all randomised patients in line with their scheduled assessments regardless of their compliance with the protocol. This allows alternative analyses to be tried and reported. (Selective reporting of these analyses is another potential source of bias to be avoided.) Plans to implement these points should be covered in the study protocol.

Anyone who follows these principles intelligently, and with a view to minimising bias, need not worry further about 'intention to treat'. Further detailed guidance can be found in a number of text-books, e.g. Pocock (1983); guidance suitable for clinical trials related to pharmaceutical development can be found in Lewis (1988).

### References

ADVANCED OVARIAN CANCER TRIALISTS GROUP (1991). Chemotherapy in advanced ovarian cancer: an overview of randomised clinical trials. *Br. Med. J.*, **303**, 884–893.

ANTURANE REINFARCTION TRIAL RESEARCH GROUP (1980). Sulfinpyrazone in the prevention of sudden death after myocardial infarction. *New Engl. J. Med.*, **302**, 250–256.

BLAKE, P. & LEWIS, J.A. (1992). Epanolol as a model for assessing patient preference in anti-anginal drug therapy. *J. Clin. Pharm.*, **32**, 85–90.

BRADFORD HILL, A. (1971). Principles of Medical Statistics (9th ed.), *Lancet*, London, p. 264.

EARLY BREAST CANCER TRIALISTS' COLLABORATIVE GROUP (1992). Systemic treatment of early breast cancer by hormonal, cytotoxic, or immune therapy. *Lancet*, **339**, 2–15 and 72–85.

FISHER, L.D., DIXON, D.O., HERSON, J., FRANKOWSKI, R.K., HEARRON, M.S. & PEACE, K.E. (1990). Intention to treat in clinical trials. In *Statistical Issues in Drug Research and Development*, Peace, K.E. (ed), pp. 331–350. Marcel Dekker: New York, p. 341.

FOOD AND DRUG ADMINISTRATION (1988). Guideline for the format and content of the clinical and statistical sections of new drug applications. FDA, US Department of Health and Human Services; Rockville, MA, USA.

FRANCE, L.A., LEWIS, J.A. & KAY, R. (1991). The analysis of failure time data in cross-over studies. *Statistics in Medicine*, **10**, 1099–1113.

FRIEDMAN, L.M., FURBERG, C.D. & DEMETS, D.L. (1982). *Fundamentals of Clinical Trials* (2nd ed.). John Wright PSG; Littleton, MA, USA, pp. 244, 246.

GAIL, M.H. (1985). Eligibility exclusions, losses to follow-up, removal of randomised patients and uncounted events in cancer clinical trails. *Cancer Treat. Rep.*, **69**, 1107–1113.

GILLINGS, D. & KOCH, G. (1991). The application of the principle of intention-to-treat to the analysis of clinical trials. *Drug. Inf. J.*, **25**, 411–424.

GOULD, A.L. (1980). A new approach to the analysis of clinical drug trials with withdrawals. *Biometrics*, **36**, 721–727.

LEWIS, J.A. (1988). Statistical standards for protocols and protocol deviations. *Recent Results in Cancer Research*, **111**, 27–33.

MEDICAL RESEARCH COUNCIL LUNG CANCER WORKING PARTY (1992). A Medical Research Council (MRC) randomised trial of palliative radiotherapy with two fractions or a single fraction in patients with inoperable non-small-cell lung cancer (NSCLC) and poor performance status. *Br. J. Cancer*, **65**, 934–941.

MURRAY, G.D. & FINDLAY, J.G. (1988). Correcting for the bias caused by drop-outs in hypertension trials. *Statistics in Medicine*, **7**, 941–946.

NORDIC COUNCIL ON MEDICINES (1989). Good clinical trial practice: Nordic Guidelines, NLN Publication No 28, Uppsala, Sweden.

PETO, R., PIKE, M.C., ARMITAGE, P., BRESLOW, N.E., COX, D.R., HOWARD, S.V., MANTEL, N., MCPHERSON, K., PETO, J. & SMITH, P.G. (1976). Design and analysis of randomised clinical trials requiring prolonged observation of each patient. I. Introduction and design. *Br. J. Cancer*, **34**, 585–612.

PETO, R., PIKE, M.C., ARMITAGE, P., BRESLOW, N.E., COX, D.R., HOWARD, S.V., MANTEL, N., MCPHERSON, K., PETO, J. & SMITH, P.G. (1977). Design and analysis of randomised clinical trials requiring prolonged observation of each patient. II. Analysis and examples. *Br. J. Cancer*, **35**, 1–39.

POCOCK, S.J. (1983). *Clinical Trials, A Practical Approach*. Wiley: Chichester, p. 176.

RUBIN, D.B. (1976). Inference and missing data. *Biometrika*, **63**, 581–592.

TEMPLE, R. & PLEDGER, G.W. (1980). The FDA's critique of the Anturane Reinfarction Trial. *N. Engl. J. Med.*, **303**, 1488–1492.

SCHWARTZ, D., FLAMANT, R. & LELLOUCH, J. (1980). *Clinical Trials*. Academic Press: London.

SENN, S. (1993). *Cross-over Trials in Clinical Research*. Wiley: Chichester, p. 220.

SHERRY, S. (1980). The Anturane Reinfarction Trial. *Circulation*, **62**, 73–78.

XAMOTEROL IN SEVERE HEART FAILURE STUDY GROUP (1990). Xamoterol in severe heart failure. *Lancet*, **336**, 1–6.